# Determination of cortisol cut-off limits and steroid dynamics in the ACTH stimulation test: a comparative analysis using Roche Elecsys Cortisol II immunoassay and LC-MS/MS

**DOI:** 10.1007/s12020-024-03752-0

**Published:** 2024-03-09

**Authors:** Sema Okutan, Nanna Thurmann Jørgensen, Lars Engers Pedersen, Stina Willemoes Borresen, Linda Hilsted, Lennart Friis Hansen, Ulla Feldt-Rasmussen, Marianne Klose

**Affiliations:** 1 Department of Endocrinology and Metabolism, Copenhagen University Hospital, Rigshospitalet, Copenhagen, Denmark; 2 Department of Clinical Medicine, Faculty of Health and Medical Sciences, Copenhagen University, Copenhagen, Denmark; 3 Department of Clinical Biochemistry, Næstved, Slagelse and Ringsted Hospitals, Slagelse, Denmark; 4 Department of Clinical Biochemistry, Copenhagen University Hospital, Rigshospitalet, Copenhagen, Denmark; 5 Department of Clinical Biochemistry, Copenhagen University Hospital, Bispebjerg Hospital, Copenhagen, Denmark

**Keywords:** Cortisol, Immunoassay, LC-MS/MS, Adrenocorticotropic hormone test, Steroids, Adrenal insufficiency

## Abstract

**Purpose:**

Measurement of cortisol concentrations is method dependent. The study aimed to establish assay-specific cut-off limits for cortisol after adrenocorticotropic hormone (ACTH) stimulation, comparing Roche Elecsys Cortisol II immunoassay to liquid chromatography-mass spectrometry (LC-MS/MS), and to assess the impact of patient characteristics, estrogen containing oral contraceptives as well as relation to other adrenocortical steroid hormone dynamics.

**Methods:**

One hundred healthy participants underwent a 250 μg ACTH-test, with plasma samples analyzed using ElecsysCortI, ElecsysCortII, and LC-MS/MS. Cortisone, corticosterone, 17-OH-progesterone, dehydroepiandrosterone sulfate (DHEAS), androstenedione, and testosterone were additionally analyzed with LC-MS/MS. Cut-off limit for a normal cortisol response to the ACTH-test was defined as: 2.5th percentile–1.96 × SE.

**Results:**

ElecsysCort II measured cortisol concentrations 21% (95% CI: 19–22%) lower than ElecsysCort I. Cut-off limits for cortisol 30 and 60 min after ACTH were 426 and 485 nmol/L (ElecsysCort II) and 411 and 470 nmol/L (LC-MS/MS). Cut-offs were unaffected by gender, or body-composition. The ACTH-test resulted in significantly increased adrenocortical steroid hormones, except for decreased cortisone concentrations (both sexes), and decreased testosterone in men (1.9 nmol/L, 95% CI: 1.3–2.5). Testosterone was increased in women (0.07 nmol/L, 95% CI: 0.02–0.13).

**Conclusion:**

ElecsysCort II has high analytical performance and yields significantly lower cortisol concentrations than prior polyclonal immunoassays. This clinically relevant difference underscores the necessity for revised cut-off limits for improved diagnostic precision. Suggested 30-minute cortisol cutoff limits are 411 nmol/L (LC-MS/MS) and 426 nmol/L (ElecsysCort II). Adrenocortical steroids increased upon ACTH stimulation, except for cortisone in both sexes and testosterone in men, both of which decreased.

## Introduction

The adrenocorticotropic hormone (ACTH) stimulation test and the insulin tolerance test (ITT) are the diagnostic tests usually performed to confirm or reject the clinical suspicion of adrenal insufficiency [1, 2]. Both tests include assessment of the cortisol concentration in serum or plasma, although assessment in saliva has been suggested in recent years [3]. Cut-off limits defining insufficiency depend on test circumstances, the type of stimulation test, and not least the assay used for measurement of cortisol [4–7]. The ACTH test is a validated and safe test [2, 8, 9], and reliable in most circumstances, although a disadvantage is its inability to detect newly onset central adrenal insufficiency [8].

Most often, cortisol concentrations are measured using immunoassays performed in automated analyzers because they are reasonably priced and have a short turnaround time, which allows for the same day or day-to-day diagnosis. However, the results are largely dependent on the antibody sensitivity and specificity in the chosen assay, and a disadvantage can thus be that the antibodies used cross-react and bind to more than the intended antigen [10, 11]. Within recent years, studies have tested different 2nd generation cortisol immunoassays using more specific antibodies and suggested cortisol cut-off limits for the ACTH test ranging from 350–460 nmol/L [12–18], which is lower than the formerly recommended cut-off limit at 500–550 nmol/L [19].

As a consequence of differences in performance and standardization of immunoassays, cut-off limits for cortisol deficiency should ideally be identified for each stimulation test and assay. The 2nd generation immunoassay *Roche Elecsys Cortisol II* (ElecsysCort II) is improved by using monoclonal antibodies to identify cortisol, which has increased its specificity compared with *Roche Elecsys Cortisol I* (ElecsysCort I), which uses polyclonal antibodies [20]. As given by the manufacturer, this resulted in cortisol concentrations approximately 20% lower when measured by the new more specific immunoassay. Thus, a switch to this new and more specific assay has significant clinical implications, as it entails the need for an adjustment of the cut-off limits defining adrenal insufficiency which guide the clinicians’ decision whether to treat or not.

The aim of the study was to perform a direct method comparison between the gold standard LC-MS/MS and the 1^st^ and 2nd generation Roche cortisol immunoassays, in order to establish test and assay specific cut-off limits for the standard 250 μg ACTH test, defined in a large well characterized group of healthy participants. In addition, we aimed to address the potential impact of gender and body composition, and to assess the impact of patient characteristics, estrogen containing oral contraceptives as well as relation to other adrenocortical steroid hormone dynamics.

## Materials and methods

The participants consisted of 50 healthy men and 63 healthy women. Thirteen of the women were using oral contraceptives (OC) containing ethinyl estradiol in combination with progestins. Inclusion criteria were healthy individuals aged >16 years. Healthy participants were defined as participants with no medical conditions known to potentially affect the outcome at the time of study participation. Exclusion criteria were ongoing treatment with glucocorticoids or spironolactone, pregnancy, breastfeeding, adrenal and pituitary disease [4].

### Sample collection and handling

Participants rested 15 min before testing after inserting an indwelling catheter in a large forearm vein. An ACTH test was performed between 0800 and 1000 h, after an overnight fast, administering 250 µg iv ACTH1–24 (Synacthen; Novartis Healthcare, Copenhagen, Denmark). All participants were tested in a supine position with sampling at baseline and at 30 and 60 min. The blood samples were centrifuged, and plasma samples were stored at −80 °C until analysis. All samples from each participant were analyzed in triplicate by ElecsysCort I and II as well as LC-MS/MS for the assessment of P-cortisol concentrations. Samples were also analyzed for cortisone, androstenedione, testosterone, 17-OH-progesterone, and dehydroepiandrosterone sulfate (DHEAS) by LC-MS/MS. Forty-one participants underwent a dual-energy X-ray scan (DXA) (model XP-26/XR-46; Norland Medical Systems, Fort Atkinson, WI) for the examination of body composition of both total and regional fat mass. The DXA scan had an in-house intra-operator variation of 5%.

### Laboratory analysis

Cortisol was quantified by the two commercial immunoassays *Roche Elecsys Cortisol I* and *II* (on Cobas 8000 e-module) (Roche GmbH, Germany) and by LC-MS/MS.

Elecsys Cortisol uses electrochemiluminescent detection. ElecsysCort I has interassay CVs (Coefficients of Variability) of 4.3%, 3.8%, and 2.5% at concentrations of 101, 436 and 1095 nmol/L, respectively, while ElecsysCort II has interassay CVs of 2.1%, 1.6%, and 1.8% at concentrations of 161, 532 and 837 nmol/L [21].

The LC-MS/MS was adapted from Phenomenex Inc. (USA) application (20655) with another column and gradient and performed using a Waters TQ-S instrument, with an i-class HPLC and a Waters Cortecs T3, 2.1 × 50 mm, 1.7 µm column. The mobile phase A was water, 0.1% formic acid, and 2 mM ammonium acetate, while mobile phase B was pure methanol. The gradient was 55% phase A decreasing linearly to 20% at 1.6 min further decreasing to 1% at 1.7 min. The rinse between samples was 99% phase B for 1.3 min and back at 3 min ending at 3.8 min. The column temperature was 40 °C. The LC-MS/MS method was ESI+ with the parameters shown in Online Resource 1. The sample was prepared by mixing with 3 parts acetonitrile containing the internal standard (9,11,12,12-D4-Hydrocortison - Sigma 705594), centrifuged and 2 µL of the supernatant was injected. The method was part of an external quality control program from UK-NEQAS, and the trueness of the calibration was verified by measuring NIST SRM 921.

### Statistics

Statistical analyses were performed using SAS Enterprise Guide Version 8.3 (SAS Institute Inc., Cary, NC, USA), R Version 4.3.1 (R Foundation for Statistical Computing, Austria), and Microsoft Excel Version 16.75.2 (Microsoft Corporation, USA). P-cortisol was log Gaussian distributed and thus log-transformed before analyses. Baseline reference intervals were calculated as the 2.5th and 97.5th percentile; mean plasma cortisol ± 1.96 × SD (standard deviation). The adjusted 2.5th percentile was calculated as the 2.5th percentile-1.96 × SE (standard error of the mean) to exclude false positive cases. The cut-off limit for a normal adrenal function was defined as the adjusted 2.5th percentile of P-cortisol concentrations 30 min after Synacthen® injection. Comparison of the 2.5th percentile and adjusted 2.5th percentile of P-cortisol concentrations between sexes and between women using OC and women not using OC were evaluated with unpaired t-tests. Assay differences were demonstrated by creating histograms, Deming regression, and Bland-Altman plots with limits of agreement (LOA) defined as mean difference ±2 SD. The response to ACTH stimulation by the other six adrenal hormones was examined with linear mixed models. The association of body composition and cortisol concentrations during the ACTH test was examined with Spearman correlation and univariate regression analyses. Observations with missing values were excluded from analyses. A difference was considered significant when *P* < 0.05.

## Results

The characteristics of the participants are summarized in Table 1.

**Table 1:** Baseline characteristics of the participants

	All^a^ *N* = *100*	Men *N* = *50*	Women *N* = *50*	Women on OC *N* = *13*
Age (year)	38 (28–51)	33 (23–44)	40 (33–55)	22 (25–30)
Height (cm)	174 ± 0.1	181 ± 0	167 ± 0	173 ± 0.1
Weight (kg)	73 ± 12	80 ± 10	66 ± 10	72 ± 12
BMI (kg/m^2^)	24 ± 3	24 ± 3	24 ± 3	24 ± 3
Hip (cm)	98 ± 7	99 ± 5	97 ± 9	97 ± 7
Waist (cm)	82 (72–89)	89 (83–93)	73 (70–82)	73 (65–74)
WHR	0.83 ± 0.08	0.89 ± 0.09	0.78 ± 0.05	0.83 ± 0.08
Sys Bp (mmHg)	117 (109–125)	121 (117–129)	112 (104–120)	109 (104–113)
Dia Bp (mmHg)	72 ± 7	73 ± 7	71 ± 9	72 ± 8
Pulse (beat/minute)	60 ± 10	59 ± 12	61 ± 8	61 ± 10

Data are presented as mean ± standard deviation or median (interquartile range)

*OC* estrogen containing oral contraceptives, *BMI* body mass index, *WHR* waist-hip ratio, *Sys Bp* systolic blood pressure, *Dia Bp* diastolic blood pressure

^a^Women using OC not included

### Comparison of ElecsysCort I and II

**Fig. 1:**
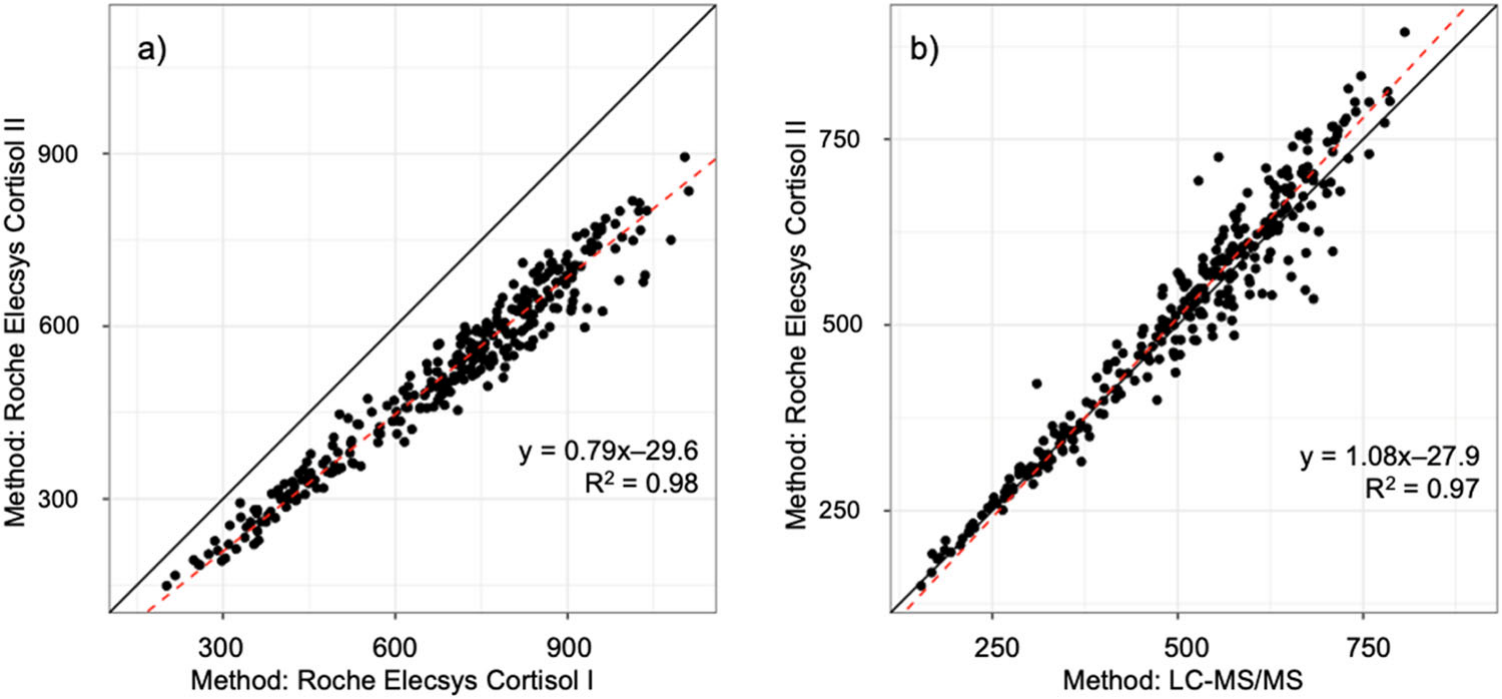
The plots show the relationship between **a** Roche Elecsys Cortisol II and I, and **b** Roche Elecsys Cortisol II and LC-MS/MS for all P-cortisol measurements from the 100 participants before and after the ACTH test. The dashed line is the equivalence between methods. The black line is the line of best fit

**Fig. 2:**
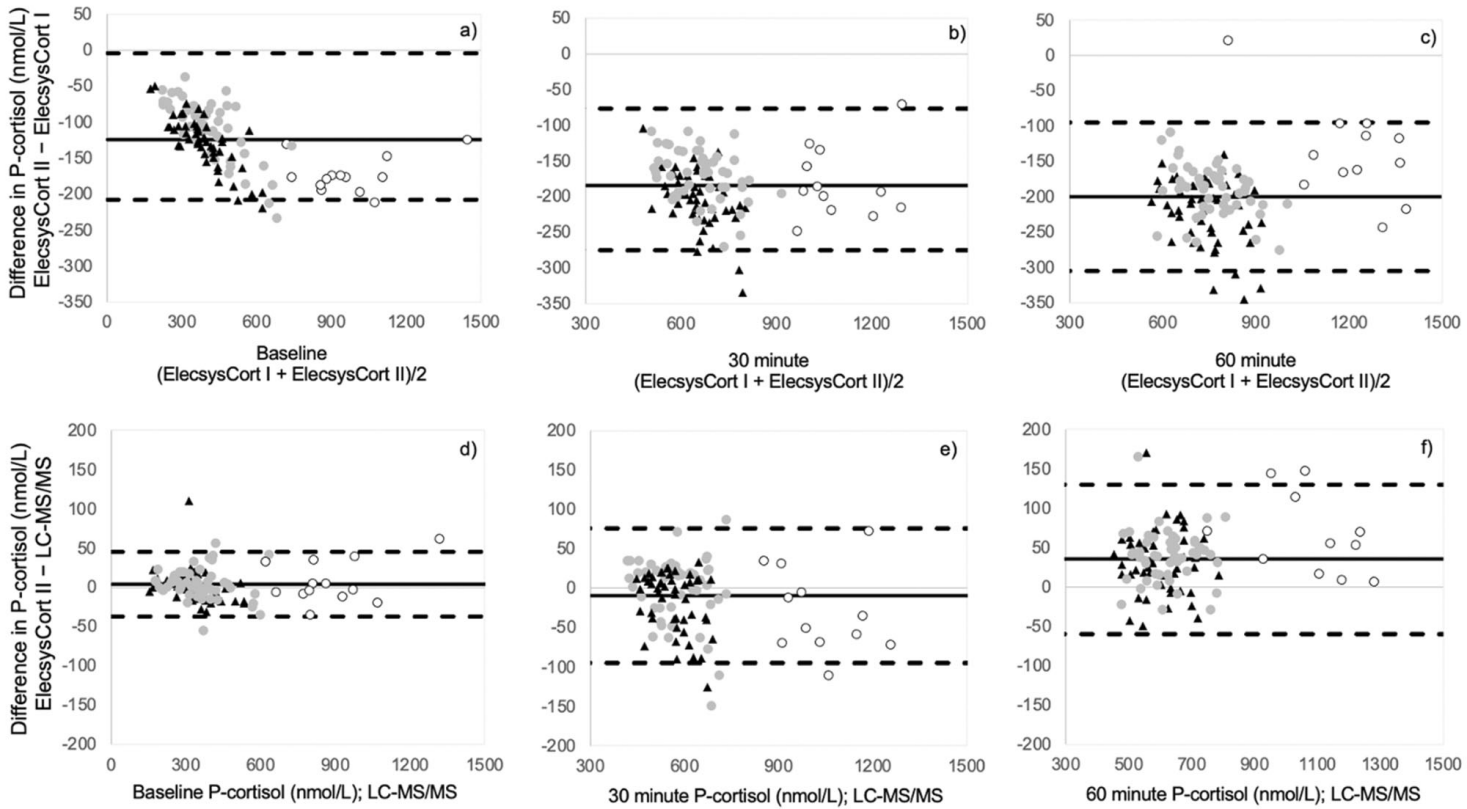
Bland–Altman plots. The first row (**a**–**c**) shows the difference between ElecsysCort I and II at baseline, 30, and 60 min after an ACTH test. The second row (**d**–**f**) shows the difference between ElecsysCort II and LC-MS/MS. For each plot the thick black line is the bias line (mean difference of the methods), and the dashed lines are the upper and lower limits of agreement (LOA, mean difference ± 1.96 × SD). A difference of 0 represents the best fit. Men; black triangles. Women; gray dots. Women on OC; white dots. OC oral contraceptives

ElecsysCort II measured P-cortisol concentrations 21% lower than ElecsysCort I as indicated by the slope of the scatter (0.79 (95% CI: 0.78–0.81)) (Fig. 1a). The agreement between the two immunoassays from Roche is illustrated in Fig. 2a–c. The Bland-Altman plots show the numeric difference in P-cortisol between the two methods which increased as the mean value of the measurements increased as indicated by the bias line (Fig. 2a–c). ElecsysCort II measured mean P-cortisol to be 124 nmol/L, 185 nmol/L, and 200 nmol/L lower than ElecsysCort I at baseline, 30 min, and 60 min, respectively.

### Comparison of ElecsycCort II and LC-MS/MS

The P-cortisol concentrations measured by ElecsysCort II and LC-MS/MS were highly concordant with a mean difference of 8% (1.08 (95% CI: 1.05–1.10)) (Fig. 1b). The numeric differences between the methods at baseline and post ACTH stimulation are illustrated in Fig. 2d–f. The numeric differences of P-cortisol concentrations exhibited less variation, although increasing with higher cortisol concentrations as indicated by the bias line. ElecsysCort II measured mean P-cortisol 9 nmol/L higher compared with LC-MS/MS 30 min post ACTH stimulation, but 4 nmol/L and 35 nmol/L lower than LC-MS/MS at baseline and 60 min post ACTH stimulation, respectively.

### Reference intervals and cut-off limits

**Fig. 3:**
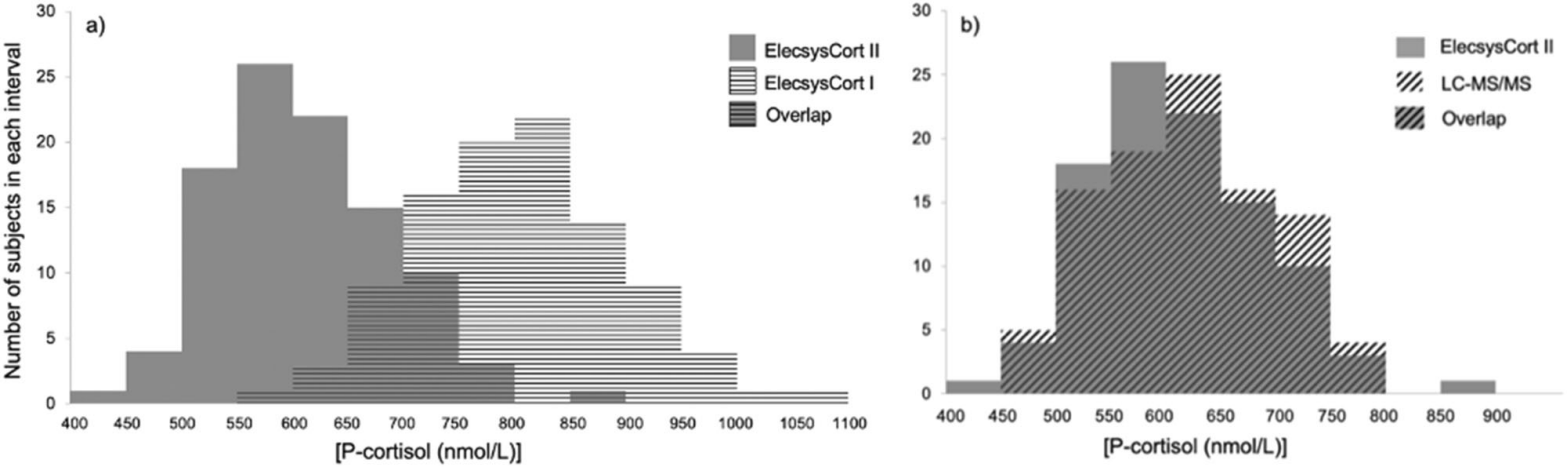
Histogram **a** illustrates the distribution of the P-cortisol concentrations from the 100 participants (women on oral contraceptives are not included) 30 min post ACTH stimulation measured with both ElecsysCort I and ElecsysCort II. The ranges are staggered. Histogram **b** illustrates the distribution of P-cortisol concentrations from the 100 participants 30 min post ACTH stimulation measured with ElecsysCort II and LC-MS/MS

The distributions of 30 min P-cortisol concentration from the 100 participants (excluding women on OC) as measured by ElecsysCort II versus ElecsysCort I (Fig. 3a) and ElecsysCort II versus LC-MS/MS (Fig. 3b).

The reference intervals and cut-off limits for normal adrenal function with each assay are shown in Table 2. The cut-off limit was 574 nmol/L with ElecsysCort I, 426 nmol/L with ElecsysCort II, and 411 nmol/L with LC-MS/MS 30 min after ACTH stimulation. There was no significant difference between the 2.5th percentiles of men and women who were not on OC (Table 3).

**Table 2:** Method related P-cortisol 95% reference intervals, 2.5th percentiles, and adjusted 2.5th percentiles before and after the ACTH test (nmol/L)

	Baseline	After 30 min	After 60 min
Reference intervals^a^			
*ElecsysCort I*	248–754	586–933	672–1080
*ElecsysCort II*	185–562	435–710	495–814
*LC-MS/MS*	169–575	426–709	479–783
2.5th percentile			
*ElecsysCort I*	248	586	672
*ElecsysCort II*	185	435	495
*LC-MS/MS*	169	426	479
Adjusted 2.5th percentile^b^			
*ElecsysCort I*	234	574	659
*ElecsysCort II*	174	426	485
*LC-MS/MS*	159	411	470

Data are in nmol/L and from all participants (*n* = 100) exclusive women on oral contraceptives (*n* = 13)

^a^Reference intervals are calculated as; mean P-cortisol ± 1.96 × SD, which gives the 2.5th and 97.5th percentiles

^b^Adjusted 2.5th percentile is calculated as; 2.5th percentile-1.96 × SE

**Table 3:** P-cortisol (nmol/L) 2.5th percentiles at baseline, 30, and 60 min after the ACTH test in men, women, and women on OC

	Men (*N* = 50)		Women (*N* = 50)			Women on OC (*N* = 13)		
	2.5th percentile^a^	Adj. 2.5th percentile^b^	2.5th percentile	Adj. 2.5th percentile	*P*-value^c^	2.5th percentile	Adj. 2.5th percentile	*P*-value^d^
Baseline								
*ElecsysCort I*	217	200	257	238	0.05	781	708	<0.0001
*ElecsysCort II*	167	155	186	172	0.10	650	578	<0.0001
*LC-MS/MS*	168	155	179	166	0.18	618	549	<0.0001
After 30 min								
*ElecsysCort I*	616	592	586	563	0.11	1064	984	<0.0001
*ElecsysCort II*	430	413	435	418	0.36	837	759	<0.0001
*LC-MS/MS*	485	439	422	398	0.11	854	774	<0.0001
After 60 min								
*ElecsysCort I*	672	646	677	651	0.41	796	722	<0.0001
*ElecsysCort II*	495	476	502	438	0.33	821	759	<0.0001
*LC-MS/MS*	479	461	480	456	0.45	750	680	<0.0001

^a^The 2.5th percentiles (in nmol/L) are calculated as; mean P-cortisol-1.96 × SD

^b^Adjusted 2.5th percentiles (in nmol/L) are calculated as; 2.5th percentile-1.96 × SE

^c^*P*-value for the difference between the 2.5th percentile of men and women

^d^*P*-value for the difference between women using oral contraceptives (OC) and women who did not

### Cortisol measurement and oral contraceptives

Women on OC (*N* = 13) had significantly higher 2.5th and adjusted 2.5th P-cortisol cut-off limits at all timestamps during ACTH stimulation regardless of the method used (Table 3).

The median increase in P-cortisol during the ACTH test from 0 to 30 min was 150 nmol/L (IQR: 119–252 nmol/L) with ElecsysCort I, 149 nmol/L (IQR: 117–237 nmol/L) with ElecsysCort II, and 192 nmol/L (IQR: 160–235 nmol/L) with LC-MS/MS. From 0 to 60 min, the median increase in P-cortisol was 316 nmol/L (IQR: 192–350 nmol/L) with ElecsysCort I, 310 nmol/L (IQR: 228–360 nmol/L) with ElecsysCort II, and 309 nmol/L (IQR: 134–386 nmol/L) with LC-MS/MS. The cortisol increase from 0–30 min was not significantly different in women taking OC compared to women who did not take OC: ElecsysCort II (mean difference 32 nmol/L, 95% CI: 11–75 nmol/L, *P* = 0.4), and LC-MS/MS (mean difference 25 nmol/L, 95% CI: 7–57 nmol/L, *P* = 0.9). nor from 0–60 min: ElecsysCort II (22 nmol/L, 95% CI: 1.5–45 nmol/L, *P* = 0.4), and LC-MS/MS (42 nmol/L, 95% CI: 0.8–83 nmol/L, *P* = 0.1).

### Influence of body composition on cortisol

Data concerning the influence of body mass index (BMI), waist-hip ratio (WHR), abdominal fat mass (ABD), and total fat mass (TFM) on baseline cortisol, stimulated cortisol, and the increase in cortisol during ACTH test as measured by LC-MS/MS are available as supplemental files (Online Resource 2, 3).

Baseline and 30- and 60-minute stimulated cortisol concentrations did not correlate with BMI, WHR, ABD, and TFM in the total group of subjects (*P* > 0.05) (see data in Online Resource 2).

In men, the increase in cortisol from 0 to 30 min correlated positively to WHR (*P* = 0.03) but was otherwise unrelated to the other body composition variables. In women, the increase in cortisol from 0 to 30 min correlated positively with WHR (*P* = 0.02), ABD (*P* = 0.001), and TFM (*P* < 0.01). These body composition variables remained significantly correlated to the increase in cortisol from 0 to 60 min (see data in Online Resource 2, 3).

### ACTH stimulation of other adrenocortical steroids

The baseline reference intervals, and increase/decrease 30 and 60 min after the ACTH test are shown in Table 4. Androstenedione, 17-OH-progesterone, and DHEAS increased significantly in both men and women after ACTH stimulation, whereas cortisone decreased in both sexes reaching significantly lower concentrations after 30 min in women (mean difference: 55 nmol/L, SD: 9.7; *P* < 0.0001) and after 60 min in men (mean difference: 57 nmol/L, SD: 7.3; *P* < 0.02). Testosterone decreased in men (mean: 1.9 nmol/L, 95% CI: 1.3–2.5; *P* < 0.0001) but increased in women (mean: 0.07 nmol/L, 95% CI: 0.02–0.13; *P* = 0.01). Delta cortisol was positively correlated with delta corticosterone (r = 0.60, *P* < 0.0001), androstenedione (r = 0.59, *P* < 0.0001), and DHEAS (r = 0.34; *P* < 0.01) in women, whereas positively correlated with corticosterone (r = 0.65, *P* < 0.0001) and androstenedione (r = 0.70; *P* < 0.0001) in men. Delta cortisol was not correlated with delta cortisone or testosterone in neither women nor men. Delta testosterone in men was positively correlated with delta DHEAS (r = 0.53; *P* < 0.0001), and delta cortisone (r = 0.31; *P* = 0.03), but not to any of the other steroid hormones. No such correlations were seen in women.

**Table 4:** Baseline and ACTH stimulated adrenocortical steroids in men and women measured with LC-MS/MS

	Baseline mean (SD)	Reference interval	*P*-value^a^	30 min mean (SD)	0–30 min increase (SE)	95% CI	*P*-value^b^	60 min mean (SD)	0–60 min increase (SE)	95% CI	*P*-value^c^
17-OH progesterone (nmol/L)											
Men	3.2 (1.9)	1.3–8.1	<0.0001	5.2 (2.6)	1.97 (0.35)	1.3–2.6	<0.0001	5.1 (2.4)	1.94 (0.34)	1.2–2.6	<0.0001
Women	1.5 (1.2)	0.34–2.6		3.8 (2.1)	2.24 (0.32)	1.6–2.9	<0.0001	3.9 (1.9)	2.4 (0.3)	1.75–2.97	<0.0001
Androstenedione (nmol/L)											
Men	2.8 (0.90)	1.6–4.8	0.2	3.9 (1.1)	1.1 (0.11)	0.88–1.31	<0.0001	4.0 (1.0)	1.2 (0.12)	0.95–1.41	<0.0001
Women	3.1 (1.6)	1.1–7.4		4.2 (1.7)	1.1 (0.11)	0.85–1.28	<0.0001	4.3 (1.7)	1.1 (0.12)	0.88–1.35	<0.0001
Testosterone (nmol/L)											
Men	17 (5.2)	10–30	<0.0001	16 (5.1)	−0.67 (0.43)	−1.35–0.22	0.057	15 (4.3)	−1.9 (0.30)	−2.5 – −1.3	<0.0001
Women	0.92 (0.57)	0.33–1.6		0.96 (0.57)	0.004 (0.023)	−0.01–0.08	0.12	0.10 (0.50)	0.07 (0.028)	0.015–0.13	0.01
DHEAS (µmol/L)											
Men	4.4 (2.0)	1.8–8.8	0.0015	4.8 (2.2)	0.48 (0.12)	0.27–0.69	<0.0001	4.7 (2.2)	0.27 (0.09)	0.085–0.45	0.005
Women	3.0 (2.2)	0.40–8.2		3.2 (2.2)	0.13 (0.44)	0.044–0.22	0.004	3.2 (2.4)	0.21 (0.053)	0.1–0.3	0.0003
Corticosterone (nmol/L)											
Men	13 (11)	3.1–41	0.43	62 (19)	49 (3.0)	43–55	<0.0001	68 (20)	55 (3.2)	48–61	<0.0001
Women	15 (14)	3.5–54		63 (14)	48 (1.9)	44–52	<0.0001	75 (17)	60 (2.4)	55–65	<0.0001
Cortisone (nmol/L)											
Men	61 (11)	44–84	0.74	59 (8.2)	−1.6 (1.23)	−4.1–0.85	0.2	57 (7.3)	−3.5 (1.5)	−6.5–0.47	0.02
Women	60 (11)	36–81		55 (9.7)	−5.6 (0.9)	−7.4 – −3.7	<0.0001	53 (8.3)	−7.4 (0.9)	−9.2– −5.6	<0.0001
Cortisol (nmol/L)											
Men	323 (91)	168–531	0.17	565 (66)	244 (13)	219–269	<0.0001	602 (80)	279 (14)	251–308	<0.0001
Women	351 (110)	179–596		575 (87)	225 (10)	204–245	<0.0001	625 (87)	275 (11)	252–297	<0.0001

The table shows the means of the hormones at each time point (nmol/L), the increase from 0–30 min (nmol/L), from 0–60 min (nmol/L), and the 95% confidence intervals (95% CI) of each hormone. All steroids are measured in nmol/L except DHEAS, which is in µmol/L

*SD* standard deviation, *SE* standard error

^a^*P*-value for the difference between men and women

^b^*P*-value for the difference between concentrations of the hormone in plasma at baseline and 30 min after ACTH stimulation

^c^*P*-value for the difference between concentrations of the hormone in plasma at baseline and 60 min after ACTH stimulation

## Discussion

This study compared 1^st^ and 2nd generation immunoassays ElecsysCort I, and ElecsysCort II, and the gold standard LC-MS/MS in measuring P-cortisol concentrations during ACTH stimulation in a large cohort of healthy individuals, in whom DXA was performed allowing for assessment of the potential relation to body composition. The P-cortisol concentrations measured with ElecsysCort II and LC-MS/MS were highly concordant with a mean difference of 8%, while ElecsysCort II measured P-cortisol concentrations 21% lower than ElecsysCort I. The cut-off limits for normal adrenal function (defined as the adjusted 2.5th percentile) were 574 nmol/L with ElecsysCort I, 426 nmol/L with ElecsysCort II, and 411 nmol/L with LC-MS/MS 30 min post ACTH stimulation. This data illustrates that the application of cut-off limits for adrenal insufficiency defined by first generation immunoassays can mislead to a large proportion of false positive cases when using more specific immunoassays such as ElecsysCort II or LC-MS/MS. The high concordance with the gold standard method LC-MS/MS measuring baseline and stimulated cortisol concentrations was also reported by others [14, 18, 20] and underlines that with ElecsysCort II we now have an immunoassay that produces more reliable results for the establishment of a cortisol cut-off limit. The explanation for this is the change from using polyclonal antibodies in ElecsysCort I to more specific monoclonal antibodies in ElecsysCort II, which has reduced the variability and enhanced the specificity of the immunoassay as cortisol now binds to a single site on the target antigen reducing the possibility of cross-reactivity with other substances. This is an important improvement as the use of immunoassays is more widespread than the use of LC-MS/MS. The strength of LC-MS/MS is its capability to quantify compounds with a high degree of sensitivity and selectivity based on unique mass/charge transition of each compound of interest. This method is however far more labor intensive, expensive, and not generally available.

In recent studies, the cut-off limit for a normal cortisol response to 250 μg ACTH varied from 400–440 nmol/L with ElecsysCort II [14, 17, 18] and from 400–412 nmol/L with LC-MS/MS [14, 18], which is in agreement with the results from the present study. Studies using the low-dose ACTH test, ITT, and glucagon stimulation test appeared to find lower cut-off limits ranging from 350–375 nmol/L with ElecsysCort II [12, 13, 15]. Thus, differences in cut-off limits should also be considered depending on stimulation tests and not only by assay variations [1, 5]. However, opinions were divided as older studies suggested the use of ITT, low-dose, and standard-dose ACTH to be equal and some even recommended low-dose ACTH test as standard method in screening for adrenal insufficiency [2, 6, 22, 23]. However, the low-dose ACTH test could be influenced by technical details e.g., loss of ACTH through tubing hereby decreasing the accuracy and specificity [24].

As anticipated, women on OC had significantly higher P-cortisol concentrations compared to women not on OC both before and after ACTH stimulation [4], due to estrogen induced elevation of cortisol binding globulin in plasma which challenges the diagnosis in OC users [10]. The increase in cortisol during ACTH stimulation was parallel to that observed in non-OC users and it could be speculated if the increase in P-cortisol during stimulation could be used to suggest or exclude adrenal insufficiency in this challenging situation. Such an approach should eventually take the baseline concentration into account as a lower increase was seen with higher baseline concentration in healthy subjects not on OC [4, 7].

Body composition did not affect baseline, 30- and 60-min stimulated cortisol concentrations during the ACTH test in men and women, which is in accordance with previous data [25, 26]. Another study found that obese men had the same increase in cortisol to ACTH stimulation compared with a normal weight control group which supports our findings [27]. On the other hand, we found that women with higher WHR and ABD had a higher increase in cortisol during the ACTH stimulation, which was also described by others [28].

The adrenocortical steroids increased upon ACTH stimulation, except for testosterone in men and cortisone in both sexes. The rise in 17-OH progesterone in women is inconclusive due to the lack of information on blood sample timing with the menstrual cycle [29]. Men experienced a significant decrease in testosterone, which is probably caused by the excessive presence of ACTH that can interfere with the usual signaling by luteinizing hormone, leading to a partial yet definite decrease in androgen production in the testes [30]. The significant decrease in cortisone, the inactive form of cortisol, may be linked to the enzyme 11-beta hydroxysteroid dehydrogenase restoring cortisone to biologically active cortisol [31]. Similarly, increased ratio of cortisol to cortisone was seen in acute-phase response to increasing CRP [32] and in intensive care patients [33].

A limitation of this study was the number of participants, albeit the number of healthy subjects assessed by ElecsysCort II was the largest so far. Achieved cut-off limits would always be more reliable if more participants were included. However, studies that included fewer or slightly more participants (ranging from 39 to 137) to define the cut-off limit for cortisol with ElecsysCort II or LC-MS/MS found very similar cut-off limits as this study [13, 14, 17, 18] supporting robustness of the suggested cut-offs. The evidence for such robustness ensures that data can be compared across centers to a larger extent than previously. The definition of a cut-off for adrenal insufficiency is historically defined based on healthy individuals’ ability to increase cortisol during i.e. surgical stress, and this response was later translated into the response of the ACTH test. Studies performed in patients with pituitary disease [17] supported the cut-off threshold including patients judged as adrenal sufficient by the treating physicians.

The development of high-performance assays allows for a more valid comparison of results across centers using different assays. As it stands now, the newer assays on the market are becoming more uniform in the measurement of cortisol in blood samples. Some of the frequently used cortisol immunoassays have been documented to measure similar P-cortisol cut-off limits (30 min post ACTH stimulation) as ElecsysCort II e.g., 423 nmol/L with Immulite 2000 by Siemens [14], 427 nmol/L with Access by Beckman Coulter [18], and 364 nmol/L with Architect Cortisol by Abbott [16]. This makes the interpretation of cortisol more consistent regardless of the assay used and the test results easily commutable when the patients are being transferred from one hospital to another. A suggestion for future guidelines in this area would be to define cortisol cut-off limits based on a specific assay combined with a specific stimulation test and a specific laboratory and including cortisol insufficient patients for comparison. However, several factors contribute to variations in results across laboratories. These include differences in assay types, often inadequate cohort sizes, variations in cohort compositions, unexplained technical disparities among laboratories, and various interfering factors such as discrepancies that may arise in methods of measurement and correction for binding proteins. Notably, such cross-laboratory comparisons, especially for different cortisol measurement methods and stimulation techniques, can potentially be detected through participation in quality control programs.

In conclusion, ElecsysCort II has a high analytical performance and measures significantly lower cortisol concentrations compared to the previous polyclonal immunoassay ElecsysCort I, but similar concentrations compared to LC-MS/MS. This difference is clinically relevant, and cut-off limits must be changed to improve diagnostic precision. We recommend a 30 min cortisol cutoff of 411 nmol/L (LC-MS/MS) and 426 nmol/L (ElecsysCort II). Cut-of limits were unaffected by gender and body composition. Whether delta cortisol can be used to suggest adrenal sufficiency warrant more data. As anticipated several adrenocortical steroids increased upon ACTH stimulation, except for testosterone in men and cortisone in both sexes which decreased.

### Supplementary information

Online Resource 1

Online Resource 2

Online Resource 3
