# Supplementary material for: Determination of cortisol cut-off limits and steroid dynamics in the ACTH stimulation test: a comparative analysis using Roche Elecsys Cortisol II immunoassay and LC-MS/MS
Source: Endocrine. 2024 Mar 9;85(1):321–30. doi: 10.1007/s12020-024-03752-0 (PMC11246257; doi:10.1007/s12020-024-03752-0)
Supplement: Supplementary file 1 — Online Resource 1 [file 12020_2024_3752_MOESM1_ESM.pdf]

Article title: Determination of cortisol cut-off limits and steroid dynamics in ACTH stimulation (Synacthen®) test: A comparative analysis using Roche Elecsys Cortisol II immunoassay and LC-MS/MS

Journal name: Endocrine (Springer)

Author names: Sema Okutan[1,2], Nanna Thurmann Jørgensen[1,2], Lars Engers Pedersen[3], Stina Willemoes Borresen[1], Linda Hilsted[4], Lennart Friis Hansen[3,5], Ulla Feldt-Rasmussen[1,2], Marianne Klose[1]

Affiliations:

[1]Department of Endocrinology and Metabolism, Copenhagen University Hospital, Rigshospitalet, Copenhagen, Denmark

[2]Department of Clinical Medicine, Faculty of Health and Medical Sciences, Copenhagen University, Copenhagen, Denmark

[3]Department of Clinical Biochemistry, Næstved, Slagelse and Ringsted Hospitals, Slagelse, Denmark

[4]Department of Clinical Biochemistry, Copenhagen University Hospital, Rigshospitalet, Copenhagen, Denmark

[5]Department of Clinical Biochemistry, Copenhagen University Hospital, Bispebjerg Hospital, Copenhagen, Denmark

Corresponding author's e-mail address: marianne.christina.klose.01@regionh.dk

Online Resource 1. The LC-MS/MS method.

	Ion	Daughter	Dwell(s)	Cone(V)	Coll(eV)
Cortisol 1	363.3	97.00	0.014	44.00	30.00
Cortisol 2	363.3	121.0	0.014	44.00	32.00
Internal standard	367.3	121.0	0.014	44.00	24.00
