# Supplementary material for: Determination of cortisol cut-off limits and steroid dynamics in the ACTH stimulation test: a comparative analysis using Roche Elecsys Cortisol II immunoassay and LC-MS/MS
Source: Endocrine. 2024 Mar 9;85(1):321–30. doi: 10.1007/s12020-024-03752-0 (PMC11246257; doi:10.1007/s12020-024-03752-0)
Supplement: Supplementary file 2 — Online Resource 2 [file 12020_2024_3752_MOESM2_ESM.pdf]

Article title: Determination of cortisol cut-off limits and steroid dynamics in ACTH stimulation (Synacthen®) test: A comparative analysis using Roche Elecsys Cortisol II immunoassay and LC-MS/MS
Journal name: Endocrine (Springer)
Author names: Sema Okutan[1,2], Nanna Thurmann Jørgensen[1,2], Lars Engers Pedersen[3], Stina Willemoes Borresen[1], Linda Hilsted[4], Lennart Friis Hansen[3,5], Ulla Feldt-Rasmussen[1,2], Marianne Klose[1]
Affiliations:
[1]Department of Endocrinology and Metabolism, Copenhagen University Hospital, Rigshospitalet, Copenhagen, Denmark
[2]Department of Clinical Medicine, Faculty of Health and Medical Sciences, Copenhagen University, Copenhagen, Denmark
[3]Department of Clinical Biochemistry, Næstved, Slagelse and Ringsted Hospitals, Slagelse, Denmark
[4]Department of Clinical Biochemistry, Copenhagen University Hospital, Rigshospitalet, Copenhagen, Denmark
[5]Department of Clinical Biochemistry, Copenhagen University Hospital, Bispebjerg Hospital, Copenhagen, Denmark
Corresponding author's e-mail address: marianne.christina.klose.01@regionh.dk

---

Online Resource 2. The Spearman correlation coefficients for the associations between the body composition variables and baseline, 30- and 60-minute stimulated P-cortisol.

	BMI	P	WHR	P	ABD	P	TFM	P
**LC-MS/MS**								
**Baseline**								
Men	-0.078	0.6	0.018	0.9	0.24	0.4	0.20	0.5
Women	-0.11	0.4	-0.066	0.7	-0.29	0.2	-0.34	0.09
**Post 30 min**								
Men	0.14	0.4	0.30	0.07	0.33	0.2	0.29	0.3
Women	0.04	0.8	0.18	0.2	0.13	0.5	-0.026	0.9
**Post 60 min**								
Men	0.10	0.5	0.21	0.2	0.33	0.2	0.27	0.3
Women	0.04	0.8	0.33	0.05	0.11	0.6	-0.1	0.8
**0 to 30 min**								
Men	0.11	0.4	0.35	0.03	0.41	0.1	0.38	0.2
Women	0.25	0.08	0.33	0.02	0.60	0.001	0.52	0.007
**30 to 60 min**								
Men	-0.077	0.6	-0.14	0.4	-0.068	0.8	-0.15	0.6
Women	0.019	0.9	0.26	0.07	-0.19	0.4	-0.17	0.4
**0 to 60 min**								
Men	0.11	0.5	0.21	0.2	0.18	0.5	0.13	0.7
Women	0.24	0.09	0.46	0.001	0.54	0.005	0.47	0.02

Online Resource 2. The table shows the Spearman correlation coefficients and P-values (P) for each body composition factor; body mass index (BMI), waist-hip ratio (WHR), abdominal fat mass (ABD), and total fat mass (TFM) with cortisol at baseline, 30- and 60-minutes after ACTH stimulation, the cortisol increase from 0-30, 30-60, and 0-60 minutes with the body composition factors; BMI, WHR, ABD, and TFM. These data do not include women on oral contraceptives.
