# Supplementary material for: Determination of cortisol cut-off limits and steroid dynamics in the ACTH stimulation test: a comparative analysis using Roche Elecsys Cortisol II immunoassay and LC-MS/MS
Source: Endocrine. 2024 Mar 9;85(1):321–30. doi: 10.1007/s12020-024-03752-0 (PMC11246257; doi:10.1007/s12020-024-03752-0)
Supplement: Supplementary file 3 — Online Resource 3 [file 12020_2024_3752_MOESM3_ESM.pdf]

Article title: Determination of cortisol cut-off limits and steroid dynamics in ACTH stimulation (Synacthen®) test: A comparative analysis using Roche Elecsys Cortisol II immunoassay and LC-MS/MS

Journal name: Endocrine (Springer)

Author names: Sema Okutan[1,2], Nanna Thurmann Jørgensen[1,2], Lars Engers Pedersen[3], Stina Willemoes Borresen[1], Linda Hilsted[4], Lennart Friis Hansen[3,5], Ulla Feldt-Rasmussen[1,2], Marianne Klose[1]

Affiliations:

[1]Department of Endocrinology and Metabolism, Copenhagen University Hospital, Rigshospitalet, Copenhagen, Denmark

[2]Department of Clinical Medicine, Faculty of Health and Medical Sciences, Copenhagen University, Copenhagen, Denmark

[3]Department of Clinical Biochemistry, Næstved, Slagelse and Ringsted Hospitals, Slagelse, Denmark

[4]Department of Clinical Biochemistry, Copenhagen University Hospital, Rigshospitalet, Copenhagen, Denmark

[5]Department of Clinical Biochemistry, Copenhagen University Hospital, Bispebjerg Hospital, Copenhagen, Denmark

Corresponding author's e-mail address: marianne.christina.klose.01@regionh.dk

---

Online Resource 3. Univariate regression analysis for the associations between the body composition variables and the delta increase in P-cortisol post ACTH stimulation from 0-30, 30-60-, and 0-60 minutes.

	BMI	P	95% CI	WHR	P	95% CI	ABD	P	95% CI	TFM	P	95% CI
0 to 30 min												
LC-MS/MS												
Men	-	-	-	421 (355)	0.24	-299-1140	-	-	-	-	-	-
Women	-	-	-	535 (214)	0.02	103-967	0.033 (0.007)	0.0002	0.018-0.048	0.0058 (0.002)	0.002	0.002-0.009
0 to 60 min												
LC-MS/MS												
Men	-	-	-	-	-	-	-	-	-	-	-	-
Women	-	-	-	784 (223)	0.002	335-1233	0.025 (0.009)	0.01	0.0055-0.044	0.004 (0.002)	0.04	0.0002-0.0085

Online Resource 3. This table shows the estimate, P-value, and 95% confidence interval for each body composition factor that correlated with cortisol in Online Resource 1. The fields marked with hyphens indicate factors that did not correlate with delta increase in P-cortisol. The significant correlations suggest a notable association when examining the increase in cortisol through univariate regression analysis, particularly evident in women and not in men.
